# Common Motor Drive Triggers Response of Prime Movers When Two Fingers Simultaneously Respond to a Cue

**DOI:** 10.3390/brainsci11060700

**Published:** 2021-05-26

**Authors:** Yasutomo Jono, Yasuyuki Iwata, Atsushi Kinoshita, Koichi Hiraoka

**Affiliations:** 1 Graduate School of Comprehensive Rehabilitation, Osaka Prefecture University, Habikino 583-8555, Japan; yasutomo-jono@naragakuen-u.jp (Y.J.); y-iwata@ncnp.go.jp (Y.I.); kinoarisu@yahoo.co.jp (A.K.); 2 Department of Rehabilitation, Faculty of Health Sciences, Naragakuen University, Nara 631-8524, Japan; 3 School of Comprehensive Rehabilitation, College of Health and Human Sciences, Osaka Prefecture University, Habikino 583-8555, Japan

**Keywords:** reaction time, motor execution process, prime mover, movement direction, common motor drive

## Abstract

This study investigated whether the motor execution process of one finger movement in response to a start cue is influenced by the participation of another finger movement and whether the process of the finger movement is dependent on the movement direction. The participants performed a simple reaction time (RT) task, the abduction or flexion of one (index or little finger) or two fingers (index and little fingers). The RT of the prime mover for the finger abduction was significantly longer than that for the flexion, indicating that the time taken for the motor execution of the finger response is dependent on the movement direction. The RT of the prime mover was prolonged when the abduction of another finger, whose RT was longer than the flexion, was added. This caused closer RTs between the prime movers for a two-finger response compared with the RTs for a one finger response. The absolute difference in the RT between the index and little finger responses became smaller when two fingers responded together compared with one finger response. Those results are well explained by a view that the common motor drive triggers the prime movers when two fingers move together in response to a start cue.

## 1. Introduction

To perform the hand movement in daily activity, a complex and cooperative activation of the hand muscles is needed. Such complex activity of the muscles has been thought to be mediated by the multi-finger synergy mechanism that allows a simple control of the muscle activities [1,2,3,4]. Despite this, the neural mechanism underlying the execution process of the multi-finger movement has not been well understood.

In the present study, the motor execution process of the multi-finger movement was speculated through observing the time taken for the prime movers to respond to a start cue, represented by the reaction time (RT). The RT involves the process of orienting to the stimulus, identifying the said stimulus, choosing the appropriate response, converting this response into the motor plan, and executing the motor plan [5]. To exclude the influence of the difference in the process other than the motor execution process on the RT, the simple RT was used in the present study. For the simple RT, orienting to the stimulus is not involved, since the same imperative cue is given at the same locus across the trials. Thus, orienting to the stimulus and the stimulus evaluation time were the same across the tasks. The motor task to be performed in response to a start cue was pre-instructed to the participants before beginning a trial block. Thus, choosing the appropriate response process and converting the response into the motor plan must have been completed before the start cue. Accordingly, those processes were also the same across the tasks in the present study. Therefore, the only differences among the tasks were the participation of the finger and movement direction, which must be the determinants of the execution process of the motor plan. Thus, the only process influencing the difference in the simple RT among the tasks was the motor execution process. The present study was conducted based on this premise.

The index finger moves independently in the force production or tapping task, but the little finger moves with the other fingers [6,7,8,9]. Previous studies using transcranial magnetic stimulation reported that the motor cortical control of the first dorsal interosseous muscle (FDI) and that of the abductor digiti minimi muscle (ADM) are different [10,11]. The FDI is the prime mover of the index finger abduction or flexion, and the ADM is the prime mover of the little finger abduction or flexion [12]. Accordingly, the motor execution process of the FDI and ADM must be different (hypothesis 1). To test this hypothesis, a comparison was made between the RT of the FDI and that of the ADM.

The motor cortical activity is dependent on the movement direction [13,14,15,16]. In the study using transcranial magnetic stimulation, corticospinal excitability of the FDI during motor imagery of the index finger flexion was greater than that during imagery of the index finger abduction [17]. Accordingly, motor cortical or corticospinal control of the finger movement is dependent on the movement direction. Based on this view, the RT, representing the time taken for the motor execution process, may be different between the flexion and abduction of the fingers (hypothesis 2). We tested this hypothesis by observing the RT between the flexion and abduction of the fingers.

It is well established that the prime movers act as synergists when performing the complex motor task [1,2,3,4]. Indeed, the limited set of synergies was activated during the masticatory motion [18]. The common fluctuation of the mean firing rates of motor units between the extensor carpi radialis longus and extensor carpi ulnaris muscles occurs, indicating a common motor drive to the motor units across the different muscles [19]. The mean firing rate curve for the vastus medialis muscle was cross-correlated with that for the vastus lateralis muscle [20]. There is motor-unit synchrony between the flexor pollicis longus and flexor digitorum profundus muscles [21]. The short-term synchronization occurs between the muscle inserted to the thumb and that inserted to the index finger [22]. The short-term synchronization reflects an event that the common motor drive is given to the motor units across the synergists [4,23]. Based on those previous findings, we hypothesized that the common motor drive triggers the two-finger response to a cue (hypothesis 3). If this view is true, then, the RT of the FDI and ADM must be closer when the two fingers respond simultaneously compared with the RT when each finger responds solely.

The abduction of the index and little fingers simultaneously occurs when opening the hand. The flexion of the index and little fingers simultaneously occurs during the power grip. Those tasks are frequently experienced in daily activity. The involvement of the synergy control mechanism for the muscles participating in a task increases with the time exposure to the task [24]. Accordingly, the prime movers of those tasks are likely mediated by the synergists’ control mechanism. The common motor drive is given to the motor units across the synergists [4,23]. Thus, the common motor drive likely triggers the response of the FDI and ADM when both the index and little fingers move in the same direction (flexion or abduction). In contrast, the task involving the flexion of one finger with the abduction of another is not often experienced in daily activity. Thus, in such a task, the synergy control of the fingers is likely less involved. Based on this view, we hypothesized that the FDI and ADM are triggered by each individual motor command when one finger moves to the flexion but other moves to the abduction simultaneously in response to a cue (hypothesis 4). If this hypothesis is true, each prime mover is triggered by an individual motor execution process, leading to a greater difference in the RT between the FDI and ADM when moving one finger in the direction of the flexion with the abduction of another vice versa in response to the cue.

## 2. Materials and Methods

### 2.1. Participants

Twelve healthy males aged 33.5 ± 1.7 years participated in this study. Only males were recruited to rule out the variability of the RT across-participants caused by sex-related influences [25]. All the participants were right-handed according to the Edinburg Handedness Inventory [26]. The participants did not have orthopedic or neurological histories. The experimental protocol was explained, and the participants gave their written informed consent to participate in this experiment. All procedures were approved by the Ethics Committee of Osaka Prefecture University (2014-107).

### 2.2. Apparatus

An oscilloscope providing visual cues was placed 150 cm in front of the participants. The visual cue was a green horizontal line on the oscilloscope. The duration of the cue was 50 ms. Ag/AgCl surface electrodes recording the electromyographic (EMG) signals were placed over the right FDI and ADM muscles with belly tendon montages. The EMG signals were amplified and bandpass filtered (15 Hz to 3 kHz) with an amplifier (MEG-2100, Nihon Kohden, Tokyo, Japan). The EMG signals were converted to digital signals at a sampling rate of 10 kHz using an A/D converter (PowerLab 800S, AD Instruments, Colorado Springs, CO, USA), and the digital signals were stored on a personal computer.

### 2.3. Procedure

The participants were seated on a table with the arms, hands, and fingers beside the trunk without any physical restriction. They moved the right finger(s) in response to a visual start cue. A warning visual cue was given 500, 600, 700, 800, 900 or 1000 ms before the start cue. The interval between the warning and visual cues was randomly assigned in each trial. The start cue was absent in 25% of the tested trials (no-go trials) to avoid a premature response in the go trials. The go trials with the RT shorter than 100 ms or longer than 500 ms were considered to be the unsuccessful go trials.

**Figure 1:**
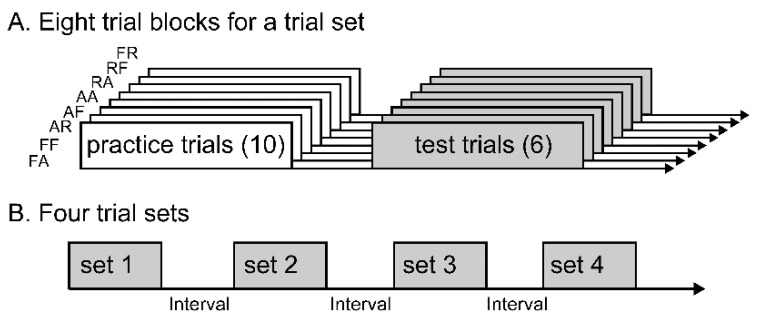
Time protocol of eight trial blocks (**A**) and four trial sets in which each set involves the eight trial blocks (**B**). Ten practice trials are followed by six successful test trials for a trial block (**A**). One of the eight tasks is randomly assigned in each trial block. This trial block is conducted for the eight tasks in each trial set. The trial set is repeated four times (**B**).

The finger movements assigned were the flexion of the index finger with the little finger at rest (FR), abduction of the index finger with the little finger at rest (AR), flexion of the little finger with the index finger at rest (RF), abduction of the little finger with the index finger at rest (RA), abduction of the index and little fingers (AA), flexion of the index and little fingers (FF), abduction of the index finger with the little finger flexion (AF) or flexion of the index finger with the little finger abduction (FA) (Table 1). The FR, RF, AR, and RA were considered to be one-finger tasks, and the FA, AF, FF, and AA were considered to be two-finger tasks. One of the eight tasks was randomly assigned in each trial block. In each trial block, the same task was repeatedly performed in response to a start cue. In each trial block, test trials were conducted until six successful go trials were obtained (Figure 1A). Ten practice trials were conducted before the test trials in each trial block to ensure that the participants were familiar with the task assigned [27]. Each trial set was composed of eight trial blocks. The participants performed four trial sets with approximately 30 s of the intervals (Figure 1B). Giving trial sets with intervals were to avoid fatigue. Taken together, 24 successful go trials were obtained in each task. The reason that we obtained 24 successful trials for calculating the mean RT was that 20 trials were a typical number to determine the mean RT in recent studies [28,29,30].

### 2.4. Data Analysis

**Figure 2:**
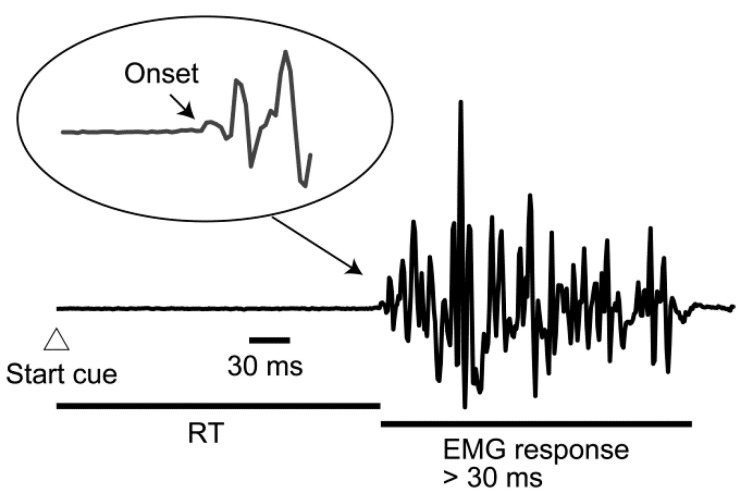
The determination of the EMG onset via visual inspection. This trace is an example of the EMG response in the experiment. The onset of the EMG response is determined on the basis of the earliest rise or decline in EMG activity beyond the steady state for the earliest EMG response that continued for more than 30 ms.

The RT in the successful go trials was calculated. The RT was defined as the time between the start cue and the EMG onset. In previous studies, the RT has been determined by visual inspection [31,32,33,34,35,36]. It has been shown that the automatic detection of the EMG onset is inaccurate and visual inspection is recommended [37]. Thus, in the present study, the EMG onset was determined by visual inspection. The onset of EMG activity was determined on the basis of the earliest rise or decline in EMG activity beyond the steady state [38]. The onset was measured for the earliest EMG response that continued for more than 30 ms. This analysis was conducted by an experimenter in all trials across the participants. The intra-rater reliability of the visual determination of the RT is so high (ICC 3, 1 = 0.992) that this analysis is reliable [39]. Thus, visual inspection of the RT conducted in the present study is reliable. An example of the determination of the EMG onset is shown in Figure 2.

The muscle, in which the RT was calculated, was named “tested muscle”. The finger, in which the tested muscle was inserted to, was named “tested finger”. The muscle or finger other than the tested one was namely conditioned muscle or finger. Three-way repeated-measures analysis of variance (ANOVA) was conducted to test the effect of the tested muscle, movement direction of the tested finger, and movement of the conditioned finger on the RT (2* (tested muscle; FDI and ADM), 2* (tested finger movement direction; flexion and abduction), 3* (conditioned finger movement; flexion, abduction, and at rest).

The absolute difference (AD) in the RT between the FDI and the ADM was calculated to estimate the difference in the RT between the prime mover of the index finger response and that of the little finger response. For the two-finger task, the AD was calculated between the FDI and ADM in the AA, FF, AF, and FA tasks. For the one-finger task, the AD was calculated between the FDI-RT in the FR task and ADM-RT in the RF task (corresponding to the FF task in the two-finger task), between the FDI-RT in the AR task and ADM-RT in the RA task (corresponding to the AA task in the two-finger task), between the FDI-RT in the FR task and ADM-RT in the RA task (corresponding to the FA task in the two-finger task), and between the FDI-RT in the AR task and ADM-RT in the RF task (corresponding to the AF task in the two-finger task). For the AD, two-way repeated-measures ANOVA was conducted (4* (pair of response directions; AA, FF, AF, and FA), 2* (number of the finger participated; one finger and two fingers).

The result of Greenhouse–Geisser’s correction was reported whenever Mauchly’s test of sphericity was significant. When the ANOVA revealed a significant effect of the conditioned finger movement, a multiple comparison test (Bonferroni’s test) was conducted. Excel-Toukei 2010, version 1.13 (Social Survey Research Information, Tokyo, Japan) was used for the statistical analysis. The alpha level was 0.05. Data were presented as the mean values and the standard error of the mean.

## 3. Results

### 3.1. RT

**Figure 3:**
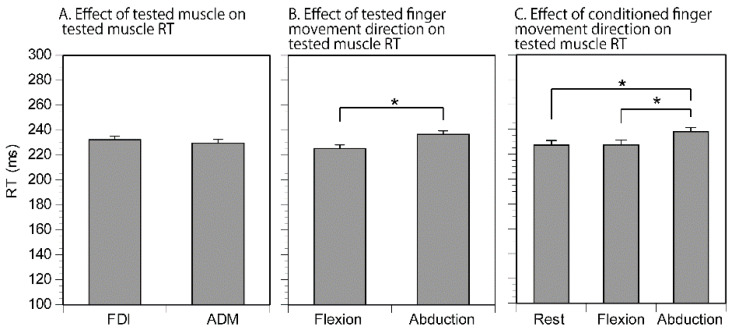
The effect of the tested muscle on the tested RT (**A**), the effect of the tested finger movement direction on the RT (**B**), and the effect of the conditioned finger movement on the RT (**C**). The bars in (**A**) represent the average RT across the tested finger movement directions and conditioned finger movements, the bars in (**B**) represent the average RT across the muscles and conditioned finger movements, and the bars in (**C**) represent the average RT across the muscles and the tested finger movement directions. Bars indicate the mean; error bars indicate the standard error. Asterisks indicate significant differences (*p* < 0.05).

The RT of the tested muscle is shown in Table 2. The average RT across the tasks was 231 ± 2 ms. There was no trial showing two or more EMG responses longer than 30 ms. The effect of the tested muscle, tested finger movement direction, and conditioned finger movement on the RT is shown in Figure 3. The three-way ANOVA failed to reveal a significant interaction among the main factors. The effect of the tested muscle was not significant (*F*(1, 11) = 2.177, *p* = 0.168, *η*^2^ = 0.165 (Figure 3A)). There was a significant effect of the tested finger movement direction (*F*(1, 11) = 10.288, *p* = 0.008, *η*^2^  = 0.483) (Figure 3B); the RT for the abduction of the tested finger was significantly longer than that for the flexion of the tested finger. The average difference in the RT between flexion and abduction was 9 ± 3 ms in the FDI and 14 ± 4 ms in the ADM. The effect of the conditioned finger movement was significant (*F*(2, 22) = 6.392, *p* = 0.006, *η*^2^  = 0.368) (Figure 3C). A multiple comparison test revealed that the RT with the abduction of the conditioned finger was significantly longer than that with the conditioned finger flexion (*t* = 3.074, *p* = 0.017, *d* = 0.50) or at rest (*t* = 3.118, *p* = 0.015, *d* = 0.51).

### 3.2. AD

**Figure 4:**
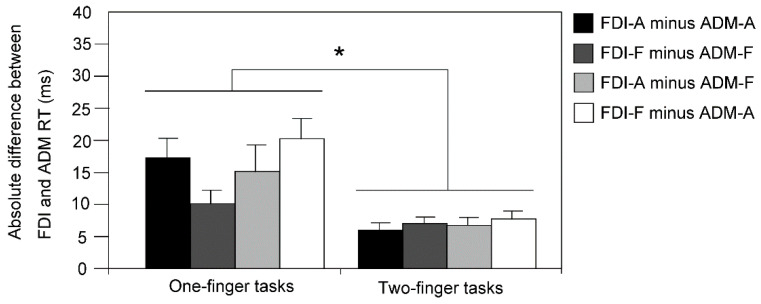
The absolute difference in the RT between the FDI and ADM (AD). Bars indicate the mean; error bars indicate the standard error. An asterisk indicates a significant difference (*p* < 0.05).

The AD of the RT is shown in Figure 4. The two-way ANOVA revealed a significant effect of the number of the finger participated (*F*(1, 11) = 17.052, *p* = 0.002, *η*^2^  = 0.608); the AD in the two-finger task was significantly smaller than that in the one-finger task. The average difference in the AD between the tasks was 9 ± 2 ms. There was no significant effect of the response direction pair (*F*(3, 33) = 2.084, *p* = 0.121, *η*^2^  = 0.159). There was no significant interaction between the two main effects (*F*(3, 33) = 1.612, *p* = 0.205, *η*^2^  = 0.128).

## 4. Discussion

In the present study, the RT of one- or two-finger response to a start cue was examined. The RT of the finger abduction was longer than that of the finger flexion. The RT of the one-finger response was longer when the abduction of another finger was added. The AD of the RT between the FDI and ADM in the two-finger task was smaller than that in the one-finger task.

### 4.1. Muscle Specificity

The index finger moves independently, but the little finger movement is dependent on the other finger movements [6,7,8,9]. The corticospinal control is different between the FDI, which is the prime mover of the index finger movement, and the ADM, which is the prime mover of the little finger [10,11]. Moreover, the enslaved response of the FDI induced by the little finger abduction is different from that of the ADM induced by the index finger abduction [40]. Based on those previous findings, we hypothesized that the motor process of the FDI and that of the ADM are different (hypothesis 1). As far as we know, the present study is first to make a comparison between the RT of the FDI and that of the ADM. The RT was not significantly different between the muscles, indicating that hypothesis 1 was not supported. Thus, the time taken to execute the response is not greatly different between the FDI and ADM, even the control mechanism is different. In daily activities, the index and little fingers move simultaneously, e.g., power grip for flexion or opening the hand for abduction. Thus, this finding is explained by a view that the time taken to execute the FDI and ADM responses are similar so that the muscles respond simultaneously to perform motor tasks in daily activities.

### 4.2. Response Direction

The motor cortical activity is dependent on the movement direction [13,14,15,16]. The corticospinal excitability of the FDI during motor imagery of the index finger flexion was greater than that during imagery of the index finger abduction [17]. Accordingly, control of the finger movement is dependent on the movement direction. Based on this view, we hypothesized that the difference in the motor process between the flexion and abduction of the finger is represented by the RT (hypothesis 2). The RT of the tested finger abduction was significantly greater than the tested finger flexion, supporting this hypothesis.

The most likely explanation of the finding is the participation of the extrinsic muscles for the flexion of the finger(s) causing involvement of the common motor drive for the motor execution of the intrinsic muscles (i.e., FDI and/or ADM). The flexion of the index or little finger is achieved not only by the intrinsic muscle but also by the extrinsic muscles [12]. Short-term synchrony of the motor units is present for the extrinsic muscles but is absent for the intrinsic muscles [41]. Accordingly, the motor units of the intrinsic muscles are under the individual control of the supraspinal centers, but extrinsic muscles receive the common motor drive (for a review, see [24]). Thus, the faster response of the finger flexion may be explained by a view that the common motor drive triggered not only the extrinsic muscles but also the intrinsic muscles when performing the flexion of the finger(s), leading to the efficient and faster motor execution process of the intrinsic muscles (i.e., FDI and/or ADM).

### 4.3. Number of Fingers Participated

The short-term synchronization of the muscle inserting to the thumb and that inserting to the index finger occurs [22,23]. The short-term synchronization of the motor units innervating one muscle and that innervating another is due to the common motor drive given to the motor units across the synergists (for a review, see [4,23]). Based on those previous findings, we hypothesized that such a common motor drive may be given to the FDI and ADM when those muscles simultaneously respond to a start cue. If this view is true, then, the RTs of the FDI and ADM become closer when two fingers respond together compared with the RTs between those muscles when one of the two fingers responds solely (hypothesis 3). A smaller AD in the RT between the FDI and ADM in the two-finger task compared with the one-finger task supported this hypothesis.

The present finding supports a view that the common motor drive is given to the prime movers when two fingers respond to a cue. This mechanism is efficient when two fingers respond together since only one motor process (common motor drive) triggers the response of the two prime movers. In contrast, if each finger responds individually, then, each individual motor execution process must be loaded for each one-finger response. Taken together, the common motor drive of the two prime movers is a reasonable explanation for the efficient motor control of the two-finger response causing closer RTs of two movers.

The RT of the mover of the tested finger movement was significantly longer when the abduction of the conditioned finger was added, but adding the flexion of the conditioned finger did not increase the RT of the tested muscle. The average RT in the tasks with one-finger abduction was 232 ms (see Table 2). This RT was longer than the RT of the task in which the finger abduction was not involved (215–227 ms). Thus, to execute the simultaneous response of two fingers involving the abduction of the finger, the time taken to execute the abduction of one finger must be shorter or that to execute the flexion of another must be longer. The present findings indicated the latter case. That is, the time taken for the execution of the finger flexion became longer for the two-finger task in which the abduction of the finger was involved, so that the FDI and ADM respond simultaneously. This view is supported by a finding that the RT in the tasks involving the finger abduction was always around 230 ms, which was similar to the RT in one-finger tasks with abduction (RA and AR tasks; see Table 2). In contrast, adding the flexion of the conditioned finger did not change the RT of the tested muscle, since the RT of the muscle for the finger flexion was short enough to respond simultaneously with the other muscle without change in the RT. Taken together, synchronization of the execution time for the simultaneous two muscle responses is likely achieved through elongating the time taken to execute the response of the mover, which is faster than the abduction response when responding with one finger. The common motor drive to the muscles may play a role for this synchronization of the onset time of those two muscle responses.

### 4.4. Task Complexity

The task complexity is structured by various dimensions (for a review, see [42]). In the present study, the dimension of the task complexity was graded by the quantity of action, i.e., one-finger or two-finger response. Such grading of action quantity has been conducted in a previous study [43]. In this pioneer study, a simple finger movement without an arm movement was compared with a simple finger movement with an arm movement, and the RT was longer for the latter task. In the present study, adding the abduction response of the conditioned finger caused a longer RT, but such effect was absent when the flexion response was added. Those mixed findings indicate that the difference in the RT between one-finger and two-finger tasks is not well explained by task complexity. The increase in the RT by adding the finger flexion is possible to be explained by the task complexity effect, but no significant change in the RT when the finger abduction is added is explained by this effect. Rather, as discussed in the Number of Fingers Participated Section, the common motor drive that triggers muscle responses simultaneously, is the likely mechanism underlying the mixed findings.

The other dimension of the task complexity graded in the present study was consistency of the finger direction in the two-finger task. The FA and AF tasks seem to be more complex tasks compared with FF or AA since the movement direction is different between the two fingers in the FA and AF tasks. If this view is true, then, a significant interaction between the effect of the tested finger movement direction and conditioned finger movement on the RT must be present. Then, the simple main effect of the tested finger movement direction over each conditioned finger movement and that of the conditioned finger movement over each tested finger movement direction must be significant. The present finding did not support this view, since there was no significant interaction between the main effects. This negative finding may be explained by a view that the time taken for the motor execution does not reflect the grading of the task complexity by manipulating the consistency of the finger movements. In most previous studies, the complexity of motor task is graded by the number of the motor components sequenced in a task or number of the body parts participated [42,43]. Thus, the present finding is novel to show that the task complexity graded by the consistency of the two finger movement directions does not influence the time taken to execute the motor response.

An alternative explanation we have to consider of this negative finding is the weak statistical power due to the small sample size causing no statistical significance. In the present study, an experiment was conducted for 12 participants. A further experiment on the greater size of the sample may detect the significant interaction between the effect of the tested finger movement direction and conditioned finger movement on the RT.

### 4.5. Task Familiarity

The tasks, in which both index and little fingers move in the same direction (abduction or flexion), are frequently experienced in daily activity. The involvement of the synergy control mechanism for the muscles participating in a task increases with the time exposure to the task [24]. The common motor drive is one mechanism mediating the control of the synergists. Thus, we expected that the FDI and ADM are likely triggered by the common motor drive when both fingers move in the same direction, i.e., AA or FF task. In contrast, the involvement of synergy control mechanism is likely less for the tasks that the flexion of one finger with the abduction of another (FA and AF tasks) is performed, since such task is not frequently experienced in daily activity. If the involvement of the synergy control mechanism is greater for the FF and AA tasks, then, a significant interaction between the conditioned finger movement direction and tested finger movement direction on the AD must have been revealed, and then, the test of the simple main effect must have revealed significant differences in the AD between the FF and FA, between the FF and AF, between the AA and FA, and between the AA and AF. The finding did not support this hypothesis, since there was no significant interaction between the conditioned and tested finger movement directions.

## 5. Conclusions

The time taken for the motor execution in the finger abduction is longer than that in the finger flexion. This may suggest that the common motor drive triggers not only the extrinsic muscle response but also the intrinsic muscle response when performing the flexion of the finger(s), leading to the faster motor execution process. The time taken for the motor execution of one finger response is longer when the abduction of another finger is simultaneously performed. The AD between the time taken for the motor execution of the FDI response and that of the ADM response in the two-finger task was smaller than that in the one-finger task. Those findings are explained by the view that a common motor drive is given to the prime movers when two fingers respond together. A weak point of the present study is that the experimenter, who analyzed the EMG onset via visual inspection, knew the muscle to be responded to in each trial, potentially causing bias of the EMG onset determination.

## Figures and Tables

**Table 1:** Motor tasks.

Task	Finger Motion		Number of Fingers Participated	Tested Muscle	
	Index	Little		FDI	ADM
FR	F	R	One finger	✔	
AR	A	R	One finger	✔	
RF	R	F	One finger		✔
RA	R	A	One finger		✔
AA	A	A	Two fingers	✔	✔
FF	F	F	Two fingers	✔	✔
AF	A	F	Two fingers	✔	✔
FA	F	A	Two fingers	✔	✔

FDI: First dorsal interosseous muscle; ADM: Abductor digiti minimi; F: Flexion; A: Abduction; R: At rest.

**Table 2:** RT of the tested muscle.

Task	FDI (ms)	ADM (ms)
FR	227 ± 7	
AR	232 ± 8	
RF		218 ± 7
RA		232 ± 7
AA	241 ± 6	240 ± 6
FF	220 ± 7	215 ± 7
AF	237 ± 9	235 ± 9
FA	237 ± 6	238 ± 6

Mean ± standard error of mean (SE).

## Data Availability

The data presented in this study are available on request from the corresponding author. The data are not publicly available due to lack of approval by the local ethics committee.
